# Higher Blood Uric Acid in Female Humans and Mice as a Protective Factor against Pathophysiological Decline of Lung Function

**DOI:** 10.3390/antiox9050387

**Published:** 2020-05-06

**Authors:** Haruka Fujikawa, Yuki Sakamoto, Natsuki Masuda, Kentaro Oniki, Shunsuke Kamei, Hirofumi Nohara, Ryunosuke Nakashima, Kasumi Maruta, Taisei Kawakami, Yuka Eto, Noriki Takahashi, Toru Takeo, Naomi Nakagata, Hiroshi Watanabe, Koji Otake, Yasuhiro Ogata, Naoko H. Tomioka, Makoto Hosoyamada, Tappei Takada, Keiko Ueno-Shuto, Mary Ann Suico, Hirofumi Kai, Junji Saruwatari, Tsuyoshi Shuto

**Affiliations:** 1 Department of Molecular Medicine, Graduate School of Pharmaceutical Sciences, Kumamoto University, 5-1 Oe-honmachi, Chuo-ku, Kumamoto 862-0973, Japan; 158y1012.kumamoto.molmed@gmail.com (H.F.); shun.kame51@gmail.com (S.K.); hnohara09@gmail.com (H.N.); 184y3005@st.kumamoto-u.ac.jp (R.N.); 162y1011@st.kumamoto-u.ac.jp (K.M.); 137p1015@st.kumamoto-u.ac.jp (T.K.); 141p1006@st.kumamoto-u.ac.jp (Y.E.); noriki.t07@outlook.jp (N.T.); mann@gpo.kumamoto-u.ac.jp (M.A.S.); hirokai@gpo.kumamoto-u.ac.jp (H.K.); 2 Program for Leading Graduate Schools “HIGO (Health life science: Interdisciplinary and Global Oriented) Program”, Graduate School of Pharmaceutical Sciences, Kumamoto University, 5-1 Oe-honmachi, Chuo-ku, Kumamoto 862-0973, Japan; 3 Division of Pharmacology and Therapeutics, Graduate School of Pharmaceutical Sciences, Kumamoto University, 5-1 Oe-honmachi, Chuo-ku, Kumamoto 862-0973, Japan; 173y3102@st.kumamoto-u.ac.jp (Y.S.); 111p1046@st.kumamoto-u.ac.jp (N.M.); oniken@kumamoto-u.ac.jp (K.O.); 4 Center for Inflammation, Immunity & Infection, Institute for Biomedical Sciences, Georgia State University, 714 Petit Science Center, 100 Piedmont Ave SE, Atlanta, GA30303, USA; 5 Division of Reproductive Engineering, Center for Animal Resources and Development (CARD), Kumamoto University, 2-2-1 Honjo, Chuo-ku, Kumamoto 860–0811, Japan; takeo@kumamoto-u.ac.jp (T.T.); nakagata@gpo.kumamoto-u.ac.jp (N.N.); 6 Department of Biopharmaceutics, Graduate School of Pharmaceutical Sciences, Kumamoto University, 5-1 Oe-honmachi, Chuo-ku, Kumamoto 862-0973, Japan; hnabe@kumamoto-u.ac.jp; 7 Japanese Red Cross Kumamoto Health Care Center, Kumamoto, 2-1-1 Nagamine-minami, Higashi-ku, Kumamoto 861-8520, Japan; k.otake@yandex.com (K.O.); y.ogata@yandex.com (Y.O.); 8 Human Physiology and Pathology, Faculty of Pharma-Science, Teikyo University, 2-11-1 Kaga, Itabashi-ku, Tokyo 173-8605, Japan; nhtomioka@pharm.teikyo-u.ac.jp (N.H.T.); hosoyamd@pharm.teikyo-u.ac.jp (M.H.); 9 Department of Pharmacy, The University of Tokyo Hospital, Faculty of Medicine, The University of Tokyo, 7-3-1 Hongo, Bunkyo-ku, Tokyo 113-8655, Japan; tappei77@gmail.com; 10 Laboratory of Pharmacology, Division of Life Science, Faculty of Pharmaceutical Sciences, Sojo University, 4-22-1 Ikeda, Nishi-ku, Kumamoto 860-0082, Japan; kshuto@ph.sojo-u.ac.jp; 11 Global Center for Natural Resources Sciences, Faculty of Life Sciences, Kumamoto University, 5-1 Oe-Honmachi, Chuo-ku, Kumamoto, 862-0973, Japan

**Keywords:** oxidative stress, uric acid (UA), mice, lung function, sexual differences, elderly, SLC2A9/GLUT9, single nucleotide polymorphism (SNP)

## Abstract

The oxidant/antioxidant imbalance plays a pivotal role in the lung. Uric acid (UA), an endogenous antioxidant, is highly present in lung tissue, however, its impact on lung function under pathophysiological conditions remains unknown. In this work, pharmacological and genetic inhibition of UA metabolism in experimental mouse models of acute and chronic obstructive pulmonary disease (COPD) revealed that increased plasma UA levels improved emphysematous phenotype and lung dysfunction in accordance with reduced oxidative stress specifically in female but not in male mice, despite no impact of plasma UA induction on the pulmonary phenotypes in nondiseased mice. In vitro experiments determined that UA significantly suppressed hydrogen peroxide (H_2_O_2_)-induced oxidative stress in female donor-derived primary human bronchial epithelial (NHBE) cells in the absence of estrogen, implying that the benefit of UA is limited to the female airway in postmenopausal conditions. Consistently, our clinical observational analyses confirmed that higher blood UA levels, as well as the SLC2A9/GLUT9 rs11722228 T/T genotype, were associated with higher lung function in elderly human females. Together, our findings provide the first unique evidence that higher blood UA is a protective factor against the pathological decline of lung function in female mice, and possibly against aging-associated physiological decline in human females.

## 1. Introduction

Respiratory dysfunction affects quality of life and facilitates the development of noncommunicable chronic lung diseases such as chronic obstructive pulmonary disease (COPD), a condition characterized by symptoms such as increased average airspace size, decreased elasticity, and decreased forced expiratory volume in one second (FEV1)/forced vital capacity (FVC) [1]. Generally, respiratory dysfunction is associated with the accumulation of oxidative stress, a condition of imbalance between reactive oxygen species (ROS) formation and cellular antioxidant capacity due to enhanced ROS generation and/or dysfunction of the antioxidant system, indicating that decreased oxidative stress is crucial for lung homeostasis maintenance [2,3]. Consistently, some epidemiological studies showed that dietary intake of antioxidants delays the decline of pulmonary function in healthy adults [4,5].

Among the endogenous antioxidants in epithelial lining fluid (ELF), uric acid (UA) exists in higher concentrations [6] and has the potential role of scavenging ROS (e.g., singlet oxygen and hydroxyl radicals) [7,8]. Although UA is a terminal product of purine metabolism in humans and great apes, this is not the case for most mammals; human and great apes lost the enzymatic activity of urate oxidase, or uricase (Uox), which converts UA to allantoin, due to a loss-of-function mutation during evolution [9]. In consequence, UA levels in human blood are much higher than in other mammals, such as mice. UA is known as an abundant antioxidant in human blood [10,11]. In fact, it was reported that high levels of UA are beneficial for central nervous system (CNS) disorders related to oxidative damage [12,13,14]. Paradoxically, high plasma UA levels are detrimental to the host due to the ability of UA to be pro-oxidative under certain conditions, thereby increasing not only the risk of gout, but also of many oxidative stress-related diseases [15,16,17]. The UA paradox in respiratory disease, especially in COPD, is often discussed in epidemiological studies [18], but the conclusions are controversial. Horsfall et al. claimed that lower blood UA levels were associated with COPD risk [19], while others showed that higher blood UA levels were correlated with COPD exacerbation [20,21]. However, whether UA directly impacts on lung function under physiological and pathological conditions remains unclear. It is also undefined which other factors, including gender, age, alcohol consumption, obesity, and urate transporter genes regulating renal absorption of UA, e.g., SLC22A12 [22], SLC2A9/GLUT9 [23], and ABCG2 [24,25], are key factors for UA-dependent lung function.

In the present study, we utilized an experimental mouse model of COPD (C57/BL6J-βENaC-transgenic mice) [26,27] and showed that pharmacological increase of plasma UA levels improved emphysematous phenotype and lung dysfunction in female but not in male mice. In vitro cellular experiments further determined that UA significantly suppressed hydrogen peroxide (H_2_O_2_)-induced oxidative stress only in the absence of estrogen, implying that the benefit of higher UA is limited to the female airway in postmenopausal conditions. We also conducted a cross-sectional retrospective longitudinal study among Japanese people in a health program and found a novel association of higher blood UA levels with higher lung function in elderly female subjects. Moreover, genetic assaying with a UA-increasing single nucleotide polymorphism (SNP) of SLC2A9/GLUT9, or the rs11722228 T/T genotype, confirmed the impact of blood UA levels on lung function in elderly female subjects. Overall, these experimental and clinical observational analyses provide the first unique evidence that blood UA directly affects lung function in female mice under pathological conditions and in human females under aging-associated physiological conditions.

## 2. Materials and Methods

### 2.1. Animal Sources and Care

We used age-matched male and female wild-type (WT) C57/BL6J mice (Charles River Laboratories, Atsugi, Japan). All mice were maintained in the animal house at Kumamoto University in accordance with the guidelines of the animal facility center of Kumamoto University and were fed with normal chow (Oriental Yeast, Tokyo, Japan) ad libitum. All experiments were performed according to the protocols approved by the Animal Welfare Committee of Kumamoto University (No. I29-200) and all methods were performed in accordance with the relevant guidelines and regulations. Uox-KO (B6;129S7-Uoxtm1Bay/J) [28], originally obtained from The Jackson Laboratory (Bar Harbor, ME, USA), was backcrossed to C57/BL6J for at least three generations. The production of C57/BL6J-βENaC-Tg mice was previously reported [26,27]. C57/BL6J-Uox knockout (KO) mice were produced by the Center for Animal Resources and Development (CARD) in Kumamoto University using our own reported reproductive technique [29].

### 2.2. Oxonate Treatment

To increase UA concentration in the blood, 500 mg/kg oxonic acid potassium salt in solution (oxonate) (Sigma-Aldrich Japan, Tokyo, Japan) was orally administered to 7–8-week-old wild-type (WT) and C57/BL6J-βENaC-Tg female/male mice per a day for 4 or 5 weeks. Oxonate was prepared by dissolving in 0.5% (*w/v*) carboxymethyl cellulose solution. Carboxymethyl cellulose sodium salt (Nacalai Tesque, Kyoto, Japan) was dissolved in distilled water for injection (Otsuka Pharmaceutical, Tokyo, Japan) and 0.5% (*w/v*) carboxymethyl cellulose solution as vehicle was orally administered. After 4–5 weeks of treatment, pulmonary function was measured using the flexiVent system (SCIREQ, Montreal, QC, Canada). Mouse lungs and blood were collected 2–3 h after the last oxonate dosing. For the healthy control group, the vehicle was administered orally. 

### 2.3. Elastase-Induced Pulmonary Emphysema Model

WT and C57/BL6J-Uox KO female and male (12- or 13-week-old) mice were administrated intratracheally with elastase from porcine pancreas (male: 0.06 mg per mouse; female: 0.05 mg per mouse) (Sigma-Aldrich Japan) dissolved in saline (Otsuka Pharmaceutical). As control, saline was treated intratracheally. After 3 weeks, we measured the pulmonary function using the flexiVent system and then collected mouse lungs and blood.

### 2.4. Measurement of Pulmonary Mechanics and Function by flexiVent

Measurement of pulmonary mechanics (resistance, compliance, and elastance) was performed with a computer-controlled small animal ventilator flexiVent as previously described [26]. Briefly, mice were anesthetized with somnopentyl (Kyoritsu, Tokyo, Japan) (1.7–1.8 mL/kg) and then an 18-gauge needle was inserted into the trachea. Mice were mechanically ventilated by the flexiVent system. Pulmonary mechanics were measured by the forced oscillation technique. The FEV0.1/FVC ratio was also measured using the flexiVent system connected to a negative pressure reservoir. Based on the data of forced expiratory volume in 0.1 second (FEV0.1) and forced vital capacity (FVC), pulmonary functional parameter FEV0.1/FVC, or forced expiratory volume % in 0.1 seconds (FEV0.1%), was determined using the flexiVent software, enabling comparison between mice and human data.

### 2.5. Histological Analysis and the Measurement of Mean Linear Intercepts (MLI)

After we measured the pulmonary parameters of each mouse by flexiVent, the whole lung was filled with approximately 1 mL of 10% formalin neutral buffer solution (FUJIFILM Wako Pure Chemical, Osaka, Japan) and then preserved in formalin for 12 h at room temperature. Samples were then washed with phosphate buffered saline (PBS) and embedded in paraffin using the histoprocessor Histos-5 (Milestone Medical, Sorisole, Italy). The tissue samples were separated into 3 sections, cut into sections that were 6 μm thick by a rotary microtome Leica RM2125RT (Leica Biosystems, Wetzlar, Germany), and stained with periodic acid-Schiff (PAS) (Sigma-Aldrich, Tokyo, Japan) and alcian blue (pH 2.5) (Nacalai Tesque) to observe mucus-secreting goblet cells. After PAS and alcian blue staining, these samples were subjected to hematoxylin and eosin (H&E) (Sigma-Aldrich, Japan) staining to visualize in light microscopy for lung morphology. The images of 10 lung sections (3 upper, 4 middle, and 3 lower) were captured randomly to draw 6 lines (300 μm in width) by BioRevo BZ-X710 (KEYENCE, Osaka, Japan). To determine the mean linear intercepts (MLI), the number of the intersections of 6 lines and the alveolar walls were counted and divided by the total length of the line, as previously described in detail [30].

### 2.6. Blood Collection and Measurement of Plasma UA and BUN

Blood was collected from the inferior vena cava using heparinized syringes with a 27-gauge needle. Plasma samples were obtained by centrifuging at 3000 rpm at 4 °C for 15 min and preserved at −80 °C. UA and blood urea nitrogen (BUN) in plasma were measured using DRI-CHEM (FUJIFILM, Tokyo, Japan). 

### 2.7. Evaluation of Plasma Antioxidant Capacity and Oxidative Stress Level

To measure plasma reactive oxygen metabolites (ROMs) as an oxidative stress marker, plasma samples from mice were subjected to d-ROMs test (Wismer Co., Tokyo, Japan), which measured total peroxides, including hydroperoxides (ROOH), i.e., metabolites produced when active oxygen and free radicals oxidize lipids, proteins, amino acids, and nucleic acids in the body. The unit of measurement was represented by Carratelli units “CARR U” (1 CARR U = 0.08 mg H_2_O_2_/dL). To evaluate plasma antioxidant capacity, we used the biological antioxidant potential (BAP) test (Wismer Co.), a photometric test to detect reduced iron from ferric (Fe^3+^) to ferrous (Fe^2+^). The reducing power was evaluated as the antioxidant power. The results of the BAP test were presented in μmol/L of reduced iron.

### 2.8. Cell Culture

The human bronchial 16HBE14o- cell line [31] was previously generated and grown in fibronectin/bovine serum albumin (BSA)-coated dishes [32] and maintained in minimum essential medium (Sigma-Aldrich Japan). The A549 cell line was maintained in Dulbecco’s modified Eagle’s medium (DMEM) (low glucose) (FUJIFILM Wako Pure Chemical). These media were added with 10% fetal bovine serum, 100 U/mL of penicillin, and 100 mg/mL of streptomycin. Primary normal (NHBE) cells from a female donor (lot: 0000543643) were purchased from LONZA (Basel, Switzerland) and maintained according to the manufacturer’s instructions. All cells were cultured in a humidified incubator at 37 °C with 5% CO_2_. 

### 2.9. Intracellular ROS Detection Assay

ROS production was detected using 5(-and-6)-chloromethyl-2’,7’-dichlorodihydro-fluorescein diacetate acetyl ester (CM-H_2_DCFDA) (Invitrogen, Carlsbad, CA, USA) dye according to the manufacturer’s instructions. 16HBE14o-, A549 (1.0 × 10^4^ cells/well), and NHBE (3200 cells/well) cells were seeded in 96-well plates and incubated until 90% confluent. NHBE was incubated with 1 nM 17β-estradiol (FUJIFILM Wako Pure Chemical, Japan) for 24 h before reagent treatment. After washing cells with PBS, 1.7 μM CM-H_2_DCFDA was added and cells were incubated for 30 min in a CO_2_ incubator. After 30 min, the reagent was removed and 7 or 14 mg/dL of UA (Sigma-Aldrich, Japan) was added. NaOH at a concentration of 5 mM and 10 mM N-acetylcysteine (NAC) (Sigma-Aldrich Japan) were used as the negative and positive controls, respectively. After 30 min, cells were incubated with 100 or 400 μM H_2_O_2_ (FUJIFILM Wako Pure Chemical, Japan)/well for 30 min and the 2′, 7′-dichloro-fluorescein (DCF) level was measured. For longer time treatment, 16HBE14o- and NHBE cells were incubated with 7 or 14 mg/dL of UA for 48 h before cells were stained with CM-H_2_DCFDA. The DCF level was immediately measured using a fluorescence microplate reader (excitation/emission = 485 nm/535 nm), TECAN infinite M1000 (TECAN, Männedorf, Switzerland). 

### 2.10. Human Subjects

We retrospectively investigated 725 health screening program participants in the Japanese Red Cross Kumamoto Health Care Center. We excluded 90 subjects with lung or airway disease (e.g., asthma, tuberculosis, lung cancer). Furthermore, to investigate the association between UA and respiratory function in postmenopausal female subjects, we excluded 82 subjects under 50 years of age. Finally, the remaining 553 subjects were enrolled in this study. The study complied with the Declaration of Helsinki and was approved by the ethics committees of the Japanese Red Cross Kumamoto Health Care Center and the Faculty of Life Sciences at Kumamoto University (Genome No. 169). All subjects provided their written informed consent prior to enrollment in the study.

### 2.11. Measurements in Humans

The laboratory tests were performed using the standard methods of the Japan Society of Clinical Chemistry. The information regarding smoking habits and alcohol intake was obtained via face-to-face interviews with healthcare providers. Hypertension was defined as a systolic blood pressure (BP) of ≥140 mmHg, a diastolic BP of ≥90 mmHg, or a history of hypertension [33]. Dyslipidemia was defined as a value of triglycerides of ≥150 mg/dL, high-density lipoprotein cholesterol of <40 mg/dL, low-density lipoprotein cholesterol of ≥140 mg/dL, or a history of dyslipidemia [34]. Hepatic ultrasonography was used to diagnose fatty liver disease. The liver was characterized as fatty liver when the liver had areas of significantly increased echogenicity relative to the renal parenchyma, the ultrasound beam was attenuated with the diaphragm indistinct, or the echogenic walls of the portal veins were less visible [35]. The diagnosis of fatty liver disease was made by the radiographer. Genomic DNA was extracted from whole blood using a DNA purification kit (FlexiGene DNA kit; QIAGEN, Hilden, Germany). The SLC2A9/GLUT9 rs11722228 (g.9914117C > T) genotype was determined using a real-time TaqMan allelic discrimination assay (Applied Biosystems, Waltham, MA, USA) in accordance with the manufacturer’s protocol (assay no. C_1216578_10). To ensure the genotyping quality, we included DNA samples as internal controls, hidden samples of a known genotype, and negative controls (water).

### 2.12. Statistical Analyses

For quantitative analysis for the in vivo and in vitro studies of the mice, the data were presented as the mean ± SEM performed in indicated replicates and were analyzed by either Student’s *t*-test or the Tukey–Kramer test using JMP® (SAS Institute Inc., Cary, NC, USA), as indicated in each figure legend. For correlation analysis, raw data of plasma UA and the oxidative stress marker in C57/BL6J-βENaC-Tg in untreated or oxo-treated mice were subjected to Pearson’s correlation coefficient test, also analyzed by JMP. The level of significance was set at *p* < 0.05. In some comparisons, effect size estimates (*d*) were calculated with *d* = 0.2 considered as a small effect, *d* = 0.5 as a medium effect, and *d* = 0.8 as a large effect.

For our clinical analyses, categorical variables were compared using Fisher’s exact test. Parametric or nonparametric continuous valuables were compared using Student’s *t*-test or the Mann–Whitney U test, respectively. The correlation between UA and FEV1/FVC was examined using Pearson correlation analysis. The values of UA were classified into 4 groups as follows: <5 mg/dL, 5–6 mg/dL, 6–7 mg/dL, and ≥7 mg/dL for male subjects and <4 mg/dL, 4–5 mg/dL, 5–6 mg/dL, and ≥6 mg/dL for female subjects. The associations of the classified UA levels with FEV1/FVC were examined using a multiple regression analysis with calculations of the partial regression coefficients (Bs) and standard errors (SEs). The B values were adjusted for potentially confounding factors, i.e., age and body mass index (BMI) as continuous variables and the presence of hypertension, dyslipidemia, fatty liver disease, and smoking status as nominal variables. Structural equation modeling was used to perform pathway analysis to assess the effects of UA level on respiratory function. The goodness-of-fit on the structural equation modeling was evaluated based on a goodness-of-fit index (GFI) of >0.90, an adjusted GFI (AGFI) of >0.90, and a root mean square error of approximation (RMSEA) of <0.10. A value of *p* < 0.05 was considered to be statistically significant. In order to examine the accuracy of the parameters of the multiple regression models and the structural equation model, bootstrap analyses were performed using 1000 replicated datasets generated by random sampling with replacement. The structural equation modeling process and the other statistical analyses were performed using the SPSS Amos software program (version 23.0; IBM Japan, Inc., Tokyo, Japan) and the SPSS software package (version 23.0; IBM Japan, Inc.), respectively. 

## 3. Results

### 3.1. Genetic Depletion of Uricase Increases Plasma UA Levels but Does Not Affect Pulmonary Phenotype in Nondiseased WT Mice

**Figure 1:**
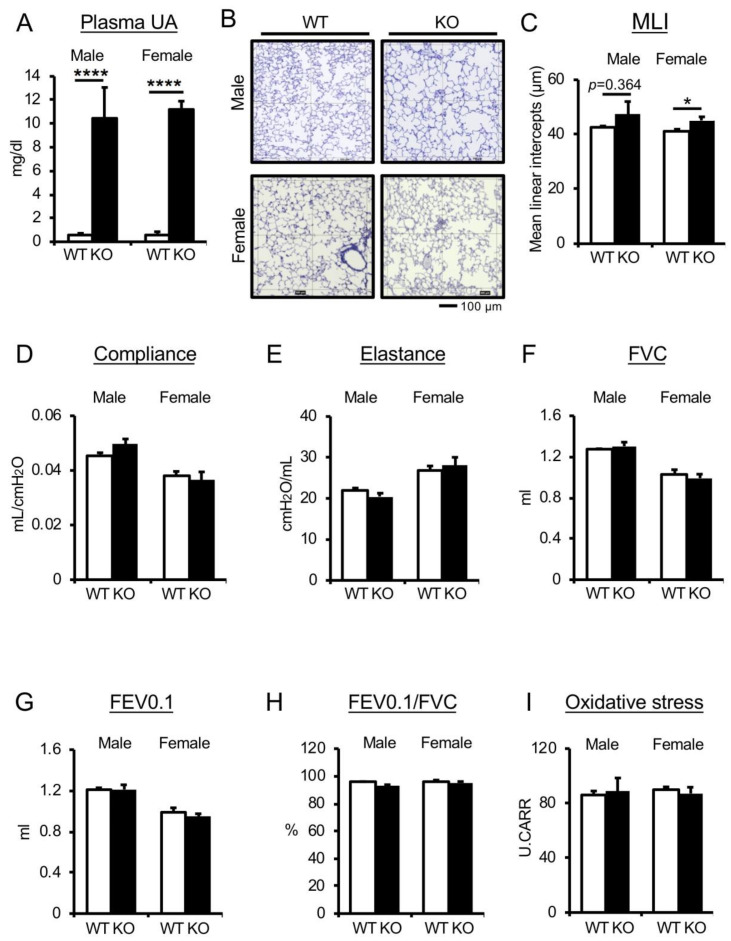
Genetic depletion of uricase does not affect pulmonary phenotype in nondiseased C57/BL6J mice. (**A**) Plasma uric acid (UA) levels (mg/d) in male and female wild type (WT) and Uox-knockout (KO) mice. *n* = 3–4 mice/group. ****p < 0.0001, versus WT; Student’s *t*-test. (**B,C**) Emphysematous phenotypes in male and female WT and Uox-KO mice. (**B**) Periodic acid-Schiff (PAS)-stained lung sections. Square diameter = 300 µm. Scale bar = 100 µm. (**C**) Quantitative morphometric analysis of alveolar septa (mean linear intercept (MLI)). *n* = 4–6 mice/group. *p < 0.05, versus WT; Student’s *t*-test. (**D–H**) Parameters of pulmonary functions are indicated (compliance, elastance, forced vital capacity (FVC), forced expiratory volume in 0.1 seconds (FEV0.1), FEV0.1/FVC). *n* = 4–6 mice/group. Compared with WT; Student’s t test. (**I**) Plasma oxidative stress levels in male and female WT and Uox-KO mice. *n* = 3–6 mice/group. Compared with WT; Student’s *t*-test. Data expressed as mean ± SEM.

Plasma UA levels in mice are notably lower than in human because mice have uricase (Uox), an enzyme that oxidizes UA to allantoin [9]. To confirm the effect of higher UA levels on pulmonary function in normal mice, we analyzed the pulmonary phenotype of Uox-knockout (Uox-KO) mice, a genetic hyperuricemia murine model [28,36]. Plasma UA levels in male and female Uox-KO mice were 10–12 times higher than those in Uox-WT (WT) mice (Figure 1A). Uox-KO mice exhibited higher plasma blood urea nitrogen (BUN) levels, a typical renal dysfunctional phenotype observed in this model (Supplementary Figure S1A). Consistently, plasma antioxidant capacity was increased in accordance with UA increase (Supplementary Figure S1B). To examine the effect of UA alteration on lung morphological and functional changes, we observed airspace enlargement and the loss of alveolar walls in lungs and measured the alveolar mean linear intercept (MLI), the most common morphometric method to evaluate emphysema in animal models (Figure 1B,C). MLI in female Uox-KO mice was significantly increased, but within the normal range, compared with nondiseased female WT mice (WT group: 41.27 ± 0.649; Uox-KO group: 45.15 ± 1.292) (Figure 1C). Pulmonary function parameters commonly used in the clinic, such as resistance (R, a level of constriction), elastance (E, the elastic rigidity), compliance (C = 1/E, a static elastic recoil pressure at a given lung volume), forced vital capacity (FVC, an overall lung volume), forced expiratory volume in 0.1 seconds (FEV0.1, an inverse indicator of bronchoconstriction), and FEV0.1% (FEV0.1/FVC, an overall lung function), were analyzed by the flexiVent system, an invasive lung-function measurement system. No differences were observed in pulmonary mechanistic markers (R, E, C) or pulmonary functional markers (FVC, FEV0.1, FEV0.1%) in male and female Uox-KO mice compared with nondiseased C57/BL6J-background mice (Figure 1D–H). To investigate the association between high UA levels and oxidative stress in the blood, we performed d-ROMs test to quantify plasma oxidative damage molecules. Plasma oxidative stress was not affected by the Uox-KO condition (Figure 1I). Taken together, the data using nondiseased WT mice confirmed that alteration of plasma UA levels had no impact on physiological lung condition in either male or female mice.

### 3.2. Genetic Depletion of Uox in Elastase-Induced COPD Mice Improves Emphysematous Phenotype in Female-Specific Manner

**Figure 2:**
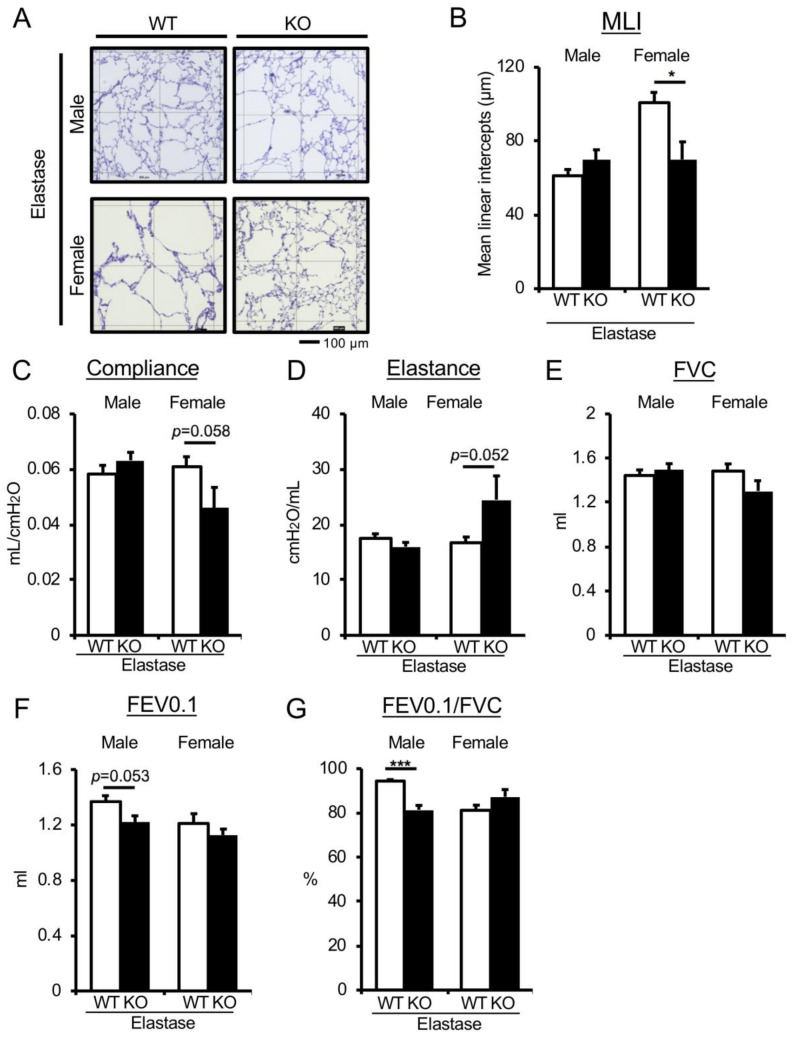
Genetic depletion of Uox in elastase-induced chronic obstructive pulmonary disease (COPD) mice improves the emphysematous phenotype in a female-specific manner. (**A**) Emphysematous phenotypes in elastase-treated male and female WT and Uox-KO mice. PAS-stained lung section. Square diameter = 300 µm. Scale bar = 100 µm. (**B**) Quantitative morphometric analysis of alveolar septa. *n* = 5–7 mice/group. *p < 0.05, versus elastase-treated WT; Student’s *t*-test. (**C**–**G**) Parameters of pulmonary function are indicated (compliance, elastance, FVC, FEV0.1, FEV0.1/FVC) in elastase-treated male and female WT and Uox-KO mice. *n* = 5–8 mice/group. ***p < 0.001, versus elastase-treated WT; Student’s *t* test. Data expressed as mean ± SEM.

To verify the effect of UA on pathological lung function decline in mice, we utilized elastase-induced acute COPD models exhibiting pulmonary phenotypes, such as emphysema and lung dysfunction, with transient increases in the expression of inflammatory and senescence markers [27]. Uox-WT (WT) and Uox-KO mice were subjected to intratracheal treatment with elastase to induce acute COPD phenotypes. Importantly, the emphysematous phenotype in female, but not in male, elastase-treated Uox-KO mice was significantly suppressed compared with WT mice (Figure 2A,B). Consistent with this result, elastase-treated female, but not male, Uox-KO mice exhibited improvement in pulmonary mechanic parameters (compliance, *p* = 0.058, effect size d: 1.31; elastance, *p* = 0.052, effect size d: 1.35), as well as slight but not statistically significant improvement in pulmonary function parameters (FVC, *p* = 0.153, effect size d: 0.95; FEV0.1/FVC, *p* = 0.144, effect size d: 0.98) compared with elastase-treated WT mice (Figure 2C–G). On the other hand, the UA increase exerted a detrimental effect in elastase-treated male Uox-KO mice (Figure 2F–G). These results suggest the idea of a female-specific benefit of UA under pathological conditions in lung dysfunction.

### 3.3. Pharmacologically Increased Plasma UA Levels in Chronic COPD Mice Reduce Oxidative Stress and Improve Emphysematous Phenotype and Lung Dysfunction in Female Mice Only

**Figure 3:**
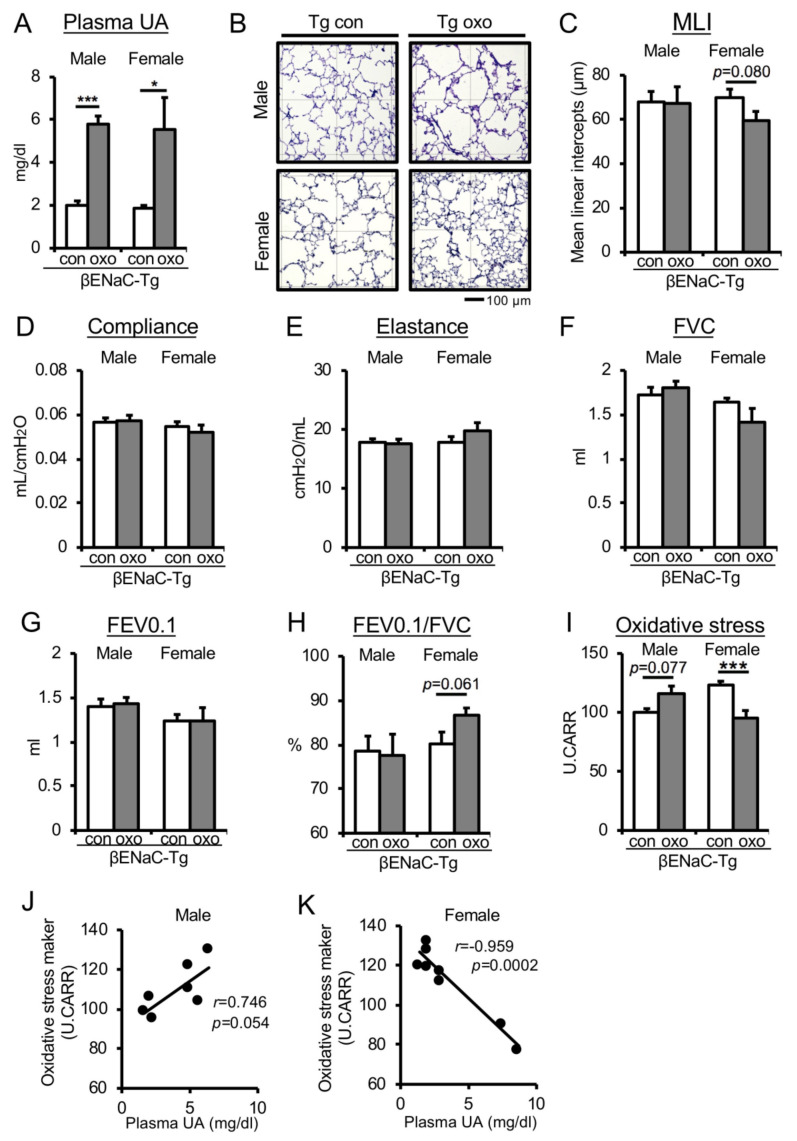
Treatment with oxonate decreases oxidative stress and improves pathophysiological lung phenotypes of COPD mice in a female-specific manner. (**A**) Plasma UA level (mg/dL) in vehicle-treated (con) or potassium oxonate (oxo)-treated male and female C57BL/6J-βENaC-Tg mice. *n* = 3–5 mice/group. *p < 0.05, ***p < 0.005, versus control C57BL/6J-βENaC-Tg; Student’s *t*-test. (**B,C**) Emphysematous phenotypes in con or oxo-treated male and female C57BL/6J-βENaC-Tg mice. (**B**) PAS and alcian blue-stained lung section. Square diameter = 300 µm. Scale bar = 100 µm. (**C**) Quantitative morphometric analysis of alveolar septa. *n* = 6–10 mice/group. Compared with control C57BL/6J-βENaC-Tg; Student’s *t*-test. (**D**–**H**) Parameters of pulmonary functions are indicated (compliance, elastance, FVC, FEV0.1, FEV0.1/FVC) in con or oxo-treated male and female C57BL/6J-βENaC-Tg mice. n = 5–8 mice/group. *p < 0.05, versus control C57BL/6J-βENaC-Tg; Student’s *t*-test. (**I**) Plasma oxidative stress levels in con or oxo-treated male and female C57BL/6J-βENaC-Tg mice. *n* = 3–6 mice/group. ***p < 0.005 versus control C57BL/6J-βENaC-Tg; Student’s *t*-test. (**J**–**K**) Correlation scatter plots of plasma UA and oxidative stress levels in con or oxo-treated male (**J**) and female (**K**) C57BL/6J-βENaC-Tg mice. Pearson’s correlation coefficient test. Data expressed as mean ± SEM.

To further verify the ameliorative effect of UA on pathological lung function decline in female mice (Figure 2), we tested a chronic COPD mouse model, C57/BL6J-βENaC-Tg mice [26,27]. We administrated the uricase inhibitor potassium oxonate (oxo), a pharmacological inducer for hyperuricemia [37], to both male and female C57/BL6J-βENaC-Tg mice (Supplementary Figure S2A). The oxo treatment increased plasma UA levels by approximately 2–3 times (Figure 3A), as well as antioxidant activity (Supplementary Figure S2B). To evaluate the effect of increased blood UA on the emphysematous phenotype of C57/BL6J-βENaC-Tg mice, we measured the MLI. Oxo treatment tended to suppress the emphysematous phenotype in C57/BL6J-βENaC-Tg female mice (*p* = 0.08, effect size d: 0.83), while there was no significant difference between the oxo-treated and control groups in C57/BL6J-βENaC-Tg male mice (Figure 3B,C). Consistently, oxo-treated female but not male C57/BL6J-βENaC-Tg mice had greater effect size values in the improvement of pulmonary mechanic parameters (E, *p* = 0.257, effect size d: 0.61) (Figure 3D–E) as well as in the pulmonary function parameter FVC (*p* = 0.213, effect size d: 0.68). Importantly, oxo treatment tended to improve FEV0.1% in female but not in male C57/BL6J-βENaC-Tg mice (*p* = 0.061, effect size d: 1.02) (Figure 3F–H). Notably, plasma oxidative stress level was significantly decreased in female mice (*p* < 0.005, effect size d: 2.23) and tended to increase in male mice (*p* = 0.077, effect size d: 1.70) (Figure 3I). Consistently, a significant negative correlation was observed between plasma oxidative stress and UA levels in female mice (*r* = −0.959: *p* < 0.001) (Figure 3J), but no correlation was observed in male mice (*r* = 0.746: *p* = 0.054) (Figure 3K). Together, in vivo study using C57/BL6J-βENaC-Tg mice also demonstrated that increased plasma UA levels improved pathophysiological lung phenotypes of COPD mice in a female-specific manner.

### 3.4. UA Suppresses Hydrogen Peroxide-Induced Oxidative Stress in Human Lung Epithelial Cells Only in the Presence of Estrogen

**Figure 4:**
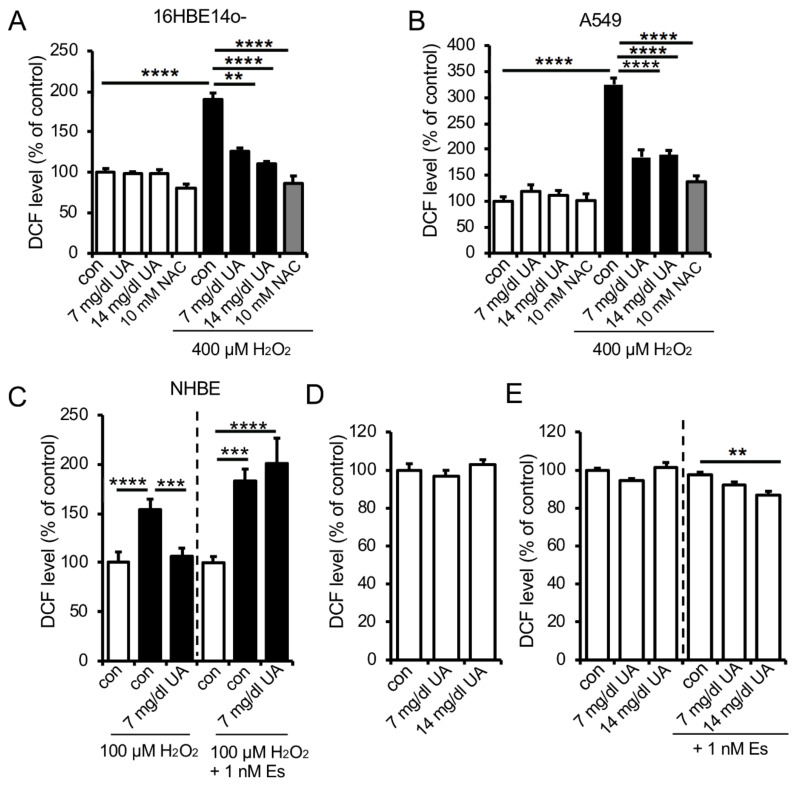
UA suppresses hydrogen peroxide (H_2_O_2_)-induced oxidative stress in human lung epithelial cells. (**A**–**C**) H_2_O_2_-induced intracellular ROS level in human lung epithelial cells treated with UA. 16HBE14o- cells (**A**) or A549 (**B**) cells were stained by CM-H_2_DCFDA (DCFDA) and treated with 7 or 14 mg/dL UA and 10 mM N-acetylcysteine (NAC) as a positive control for 30 min before incubating with 400 μM H_2_O_2_ for 30 min at 37 °C. (**C**) Female-derived primary human bronchial epithelial (NHBE) cells incubated with 1 nM 17β-estradiol for 24 h were stained by 5(-and-6)-chloromethyl-2’,7’-dichlorodihydro-fluorescein diacetate acetyl ester (DM-DCFDA) before incubating with 100 μM H_2_O_2_ for 20 min after treating with 7 mg/dL UA for 20 min at 37 °C. 2’,7’-dichlorodihydro-fluorescein (DCF) levels were measured using a fluorescent microplate reader. *n* = 4–5/group. Data are indicated as % of control and expressed as mean ± SEM. (**D**–**E**) Effects of long-term UA treatment on reactive oxygen species (ROS) production in human lung epithelial cells. (**D**) 16HBE14o- cells were stained by DCFDA after incubating with 7 or 14 mg/dL UA for 48 h at 37 °C. (**E**) Female-derived NHBE cells incubated with 1 nM 17β-estradiol in media for 48 h were stained by DCFDA and then incubated with 100 μM H_2_O_2_ for 20 min after treating with 7 mg/dL UA for 20 min at 37 °C. DCF levels were measured using a fluorescent microplate reader. *n* = 4–5/group. Data are indicated as % of control and expressed as mean ± SEM. Data are mean ± SEM; *n* =3/ group. **p < 0.01, and ***p < 0.001, ****p < 0.0001were assessed by the Tukey–Kramer test.

UA is known to exist in epithelial lining fluid (ELF) in high concentrations [10,11]. To investigate the direct role of UA in the airway, we examined the effect of UA on intracellular oxidative stress in several human lung epithelial cells. UA (7 and 14 mg/dL) significantly suppressed H_2_O_2_ (400 μM)-induced intracellular oxidative stress in the human bronchial epithelial cell line 16HBE14o- (Figure 4A), as well as in the adenocarcinoma human alveolar basal epithelial cell line A549 (Figure 4B). Because of the female-specific ameliorative effect on pulmonary function in animal models (Figure 2 and Figure 3), it can be speculated that estrogen is involved in a UA-dependent antioxidative pulmonary protective effect. To test this hypothesis, we treated primary human bronchial epithelial cells (NHBE) derived from a female donor with 17β-estradiol and measured the UA-dependent suppressive effect on H_2_O_2_-induced oxidative stress. Importantly, 7 mg/dL of UA inhibited H_2_O_2_-induced oxidative stress in the absence of 1 nM of 17β-estradiol, whereas its suppressive effect was dampened in the presence of 17β-estradiol (Figure 4C).

Finally, we also tested the possible detrimental effect of UA in airway epithelial cells caused by its pro-oxidative activities, as shown in other tissues [38,39,40]. Long-term treatment with UA did not induce ROS generation in 16HBE14o- cells (Figure 4D). Moreover, UA also did not induce ROS in primary NHBE cells treated with 17β-estradiol (Figure 4E). Overall, these in vitro cellular data indicated that UA has the potential to work as a relatively nontoxic antioxidant to specifically protect from oxidative stress.

### 3.5. Serum UA Levels Inversely Correlated with Aging-Dependent Decline of Lung Function in a Female-Specific Manner in Humans

To verify whether the protective role of UA on lung dysfunction deduced from animals and in vitro experiments was also conserved in humans, we performed an exploratory epidemiological study in a total of 553 elderly subjects who were participants in a health screening program. In the present study, we used an aging-dependent decline of lung function as the respiratory dysfunction in humans, because lung aging is associated with the accumulation of oxidative stress, a condition of imbalance between ROS formation and cellular antioxidant capacity due to enhanced ROS generation and/or dysfunction of the antioxidant system, indicating that decreasing oxidative stress is crucial for lung homeostasis maintenance [2,3]. The clinical characteristics of the subjects in males and females are shown in Table 1. The parameters for respiratory function, i.e., FEV1/FVC, %FEV1, and %FVC, were significantly higher in female than in male subjects. The frequencies of the SLC2A9 rs11722228 C/C, C/T, and T/T genotypes were 50.3%, 41.6%, and 8.1%, respectively, and existed in a Hardy–Weinberg equilibrium (*p* > 0.05). The SLC2A9 genotype frequencies were not significantly different between male and female subjects (Table 1).

**Figure 5:**
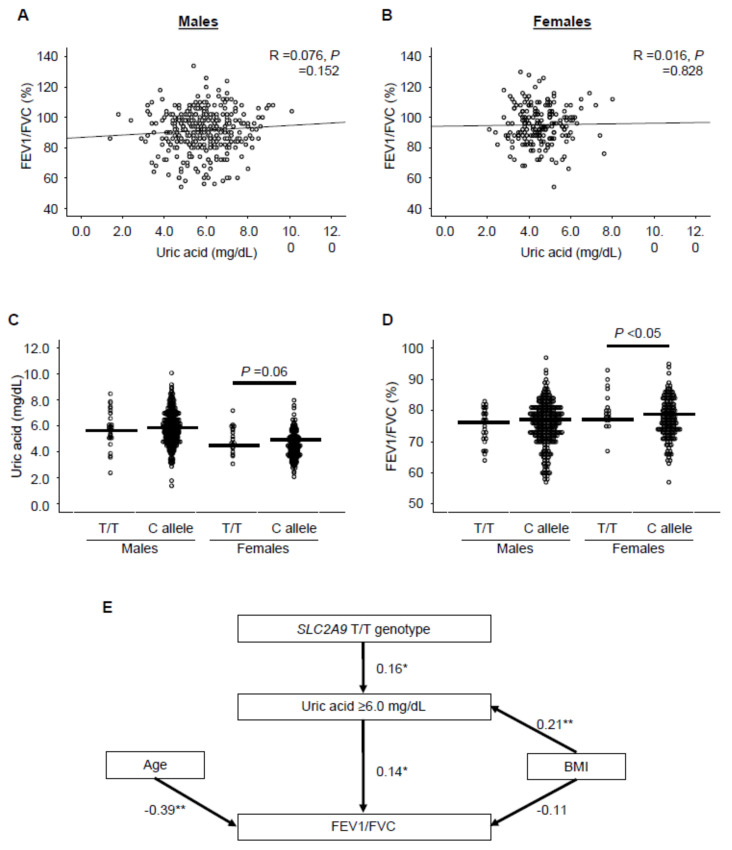
The possible association between uric acid and lung function in humans. (**A,B**) The correlations between UA and FEV1/FVC in males (**A**) and females (**B**). Correlation coefficients (r) and *p*-values were calculated by Pearson’s correlation test. (**C,D**) The association of the SLC2A9/GLUT9 genotype with uric acid (**C**) or FEV1/FVC (**D**) in males and females. P values were calculated by the Mann–Whitney U test. (**E**) Structural equation modeling diagram of respiratory function and uric acid levels in females. Lines with numbers indicate significant paths with standardized partial regression coefficients (*p < 0.05, **p < 0.01). Arrows represent an association between two factors. The β values ranged from –1 to 1, with a positive value representing a positive correlation and a negative value representing a negative correlation. Β: standardized partial regression coefficient; BMI: body mass index; FEV1: forced expiratory volume in 1 second; FVC: percent predicted forced vital capacity; SLC2A9/GLUT9: solute carrier family 2 member 9.

In the correlation analysis, no association between the UA level and FEV1/FVC was observed in both male and female subjects (Figure 5A,B). We next examined the association between the classified UA levels and FEV1/FVC using multiple linear regression analysis incorporating the subjects’ age, body mass index, hypertension, dyslipidemia, fatty liver disease, and smoking status as confounding factors (Table 2). Since the mean value, as well as 25th, 50th, and 75th percentiles, of UA levels were approximately 1 mg/dL lower in female than in male subjects (Table 1 and Supplementary Figure S3), the subjects were divided according to 1 mg/dL increases in UA level from <5 mg/dL and <4 mg/dL in males and females, respectively (Table 2). The patient characteristics in each group are shown in Supplementary Tables S1 and S2 for male and female subjects, respectively. Interestingly, the FEV1/FVC value was higher in subjects with UA ≥ 6.0 mg/dL than in those with UA < 4.0 mg/dL among female subjects; this relationship was not observed in male subjects (Table 2). We also applied bootstrap evaluation to validate the results derived from the original dataset and found that all parameters obtained using the multiple linear regression analysis were equivalent to those estimated from the bootstrap method (Table 2). 

Next, we assessed the association of the SLC2A9/GLUT9 genotypes with UA levels (Figure 5C) and FEV1/FVC (Figure 5D). In female subjects, UA levels tended to be higher in carriers of the SLC2A9/GLUT9 T/T genotype than in carriers of the C allele (Figure 5C), and the FEV1/FVC was significantly higher in carriers of the T/T genotype than in carriers of the C allele (Figure 5D). On the other hand, in male subjects, no association was observed between the SLC2A9/GLUT9 genotype and UA level or FEV1/FVC (Figure 5C,D). To verify the observed associations of respiratory function with UA ≥ 6.0 mg/dL and the SLC2A9/GLUT9 genotypes in female subjects, we constructed a structural equation model incorporating age and BMI among female subjects (Figure 5E). The GFI, AGFI, and RMSEA of the model were 0.995, 0.985, and <0.001, respectively, indicating a good fit for the structural equation model. The SLC2A9/GLUT9 T/T genotype appeared to influence the frequency of UA ≥ 6.0 mg/dL (Figure 5E). Furthermore, UA ≥ 6.0 mg/dL was associated with an increase in FEV1/FVC (Figure 5E). A high BMI was associated with an increase in the frequency of UA ≥ 6.0 mg/dL and tended to be associated with a decrease in FEV1/FVC. The parameters (e.g., standardized partial regression coefficients) did not converge when the structural equation model incorporated other potential confounding factors, such as fatty liver disease. An increase in age was independently associated with a decrease in FEV1/FVC (Figure 5E). Thus, these findings of the present human studies implied a possible inverse correlation between serum UA level and aging-dependent decline of lung function in a female-specific manner.

## 4. Discussion

UA is one of the antioxidants that is prominent in the airway mucous membrane [6], but its actual role, especially in the regulation of lung function, remains unspecified. In this study, we examined the impact of blood UA levels on lung dysfunction between genders by utilizing experimental mice model as well as human observational analyses. Overall, our findings demonstrated that UA is highly likely to be a protective factor against aging-associated physiological and COPD-associated pathological decline of lung function in female humans and mice. Because the negative aspects of UA (e.g., UA crystal formation, oxidative stress induction, etc.) have attracted attention in a variety of diseases [15,16,17], this study provides a novel concept of the beneficial aspects of UA in the field of human health.

Whether UA has epidemiologically beneficial effects on physiological and pathological lung dysfunction is controversial. Song et al. reported that higher serum UA levels were positively correlated with lung function in the middle-aged healthy population, regardless of gender [41]. Horsfall et al. showed that lower serum UA levels were related to a higher risk of COPD in a heavy smoker group in a population-based cohort study [19]. Nicks et al. reported that lower UA levels were associated with COPD severity in the cross-sectional study [42]. However, these reports did not clarify whether UA alteration directly contributes to protection against lung dysfunction. Our study focused on identifying whether experimental alteration of UA affected lung function under physiological and pathological conditions. Our data supported the other groups’ reports except for gender segregation; unexpectedly, a specific limited condition, i.e., female mice under COPD conditions, was uncovered whereby UA worked as a pulmonary protective factor. Serum UA levels in females are generally lower than in males, implying that females may have a lower antioxidant capacity in the airways, thereby inducing susceptibility to oxidative stress-induced damage. In fact, decreased antioxidant capacity is associated with age-related decline of pulmonary function [43], therefore, our data suggested that high levels of UA in elderly women exert an antioxidant effect in the lungs and maintain lung function by removing increased ROS. 

A significant increase in plasma UA levels due to Uox gene deficiency did not affect lung function in mice under normal conditions (Figure 1). Furthermore, high-dose UA treatment did not affect ROS production under normal conditions (Figure 4). On the other hand, increased UA levels improved lung function in female mice in a COPD-dependent pathological decline in lung function (Figure 2 and Figure 3), and high-dose UA treatment protected against H_2_O_2_-induced oxidative stress in human airway epithelial cells (Figure 4). However, these results did not explain the sexual difference in the response to improved lung function. In female COPD mice, plasma UA levels were significantly negatively correlated with plasma oxidative stress, but this was not observed in male mice. In the COPD model, the pulmonary phenotype in females was more severe than in males [44]. This may be attributed to the fact that the female model in the control group had a slightly higher level of oxidative stress than the male model mice. In an in vitro study using NHBE cells from a female donor, UA treatment with estrogen did not suppress oxidative stress. When estrogen is sufficient, the induction of oxidative stress is lower (Figure 4). Considering these results, UA might beneficially work only under conditions with higher oxidative stress, such as aging in postmenopausal and COPD-like conditions.

The results of our human study showed that the SLC2A9/GLUT9 rs1917760 polymorphism and UA levels were associated with respiratory function among the elderly female subjects. A previous meta-analysis of population-based cohort studies indicated that female subjects had faster rates of age-related decline in respiratory function than male subjects in a smoking population [45]. In another study, postmenopausal female subjects who used hormone replacement therapy (i.e., use of estrogen and progesterone) exhibited higher levels of FEV1 and were less likely to suffer from airway obstruction, even after adjusting for variables known to influence respiratory function [46], suggesting that female sex hormones may protect against respiratory function decline. Combining our results and these previous findings, the antioxidative effect of UA may have an important role in protecting respiratory function in elderly female individuals.

The results of structural equation modeling suggested that high BMI was associated with a protective effect of high UA levels on respiratory function among elderly females. A previous epidemiological study showed that many Japanese COPD patients were categorized as having emphysema and being underweight [47]. A recent cohort study conducted on 282,135 Korean subjects showed that underweight status was associated with respiratory function decline [48]. Moreover, the cohort study also showed that subjects with low FEV1 and FVC were more likely to have low muscle mass [48]. Since malnutritional and/or physically inactive individuals are more likely to have low muscle mass and strength, especially in elderly [49,50], we suggest that lifestyle modifications, including nutritional and/or exercise management for underweight elderly females, may provide respiratory function protection partially through increased UA levels.

Fatty liver was reported to be significantly associated with not only high serum UA levels [51,52,53] but also decreased lung function [54]. Additionally, nonalcoholic fatty liver disease occurs at a higher rate in women after menopause [55]. In this study, we analyzed the association between the classified UA levels and the FEV1/FVC using multiple linear regression analysis incorporating the presence or absence of fatty liver. Accordingly, the FEV1/FVC value was higher in individuals with UA ≥ 6.0 mg/dL than in those with UA < 4.0 mg/dL, even after adjusting for the presence or absence of fatty liver disease, but only in females (Table 2). Furthermore, we could not incorporate the fatty liver into the structural equation modeling diagram of respiratory function and UA levels in females (Figure 5E) because the parameters did not converge. As was the case with the previous studies [51,52,53], although the prevalence of fatty liver disease was greater in subjects with high UA levels than in those with low UA levels among male and female subjects (Supplementary Tables S1 and S2), age, but not fatty liver disease, was significantly associated with a decrease in the FEV1/FVC value (Supplementary Table S3). In our clinical investigation, we focused on an aging-dependent decline in lung function as the respiratory dysfunction in humans. Therefore, a large portion (37.6%) of our study subjects were elderly, i.e., >70 years old, individuals, whereas most subjects seemed to be non-elderly individuals in previous studies that showed a relationship between nonalcoholic fatty liver disease and decreased lung function [54]. Nevertheless, further studies evaluating the UA–fatty liver–sex dimorphism triangle in terms of lung function are needed in a larger population.

Although the present clinical investigations showed that UA is likely a protective factor for aging-related physiological and pathological decline in lung function among females, it should be recognized that high UA levels are related to several diseases (e.g., coronary heart disease (CHD)) and pathophysiological conditions (e.g., hypoxia). A previous meta-analysis reported that hyperuricemia appeared to increase the incidence of CHD as well as the mortality of CHD patients [56]. In addition, epidemiological studies showed that hyperuricemia was an independent risk factor for the development of chronic kidney disease (CKD) and end-stage renal disease [57]. Furthermore, serum UA levels were reported to be higher in individuals with other risk factors for CHD and CKD (e.g., hypertension, hyperglycemia, dyslipidemia, fatty liver, high BMI, alcohol consumption, and smoking) than in those without [58]. Extracellular UA is known to have antioxidant activity in terms of scavenging peroxynitrite and reacting with singlet oxygen, lipid-derived radicals, and ferric iron [59]. On the other hand, increased intracellular UA is produced by xanthine oxidoreductase (XOR) or by uptake via urate transporters, thereby activating mitogen-activated protein kinases (MAPK) and NADPH oxidase, resulting in increased mitochondrial and cytoplasmic oxidative stress, which may be implicated in metabolic diseases [59]. Intracellular UA was also reported to accumulate by increased XOR activity under hypoxic conditions [60]. Previous human studies showed that serum UA levels were increased in hypoxic conditions among patients with acute exacerbation of COPD [61], obstructive sleep apnea [62], pulmonary hypertension [63], and chronic respiratory failure [64]. Moreover, smoking under hypoxic conditions is a potent risk factor for various inflammatory diseases [65], and both acute and chronic smoking exposures increased mRNA expression and promoter activity of XOR in rats in vivo [66]. Furthermore, an epidemiological study of 8662 Japanese individuals who underwent a health checkup reported that smoking habits increased serum UA levels [67]. Therefore, whether UA acts as a protective or harmful factor for lung function may vary depending on the conditions under the presence or absence of hypoxia and smoking habits. Based on the above-stated information, when considering the relationship between UA and lung function, careful attention should be paid not only to serum UA levels but also to the background affecting the UA level (e.g., the presence of cardiovascular and renal disease, hypoxic condition, and lifestyle).

Several limitations associated with the present human study should be noted. First, the human study was a retrospective design with a small number of subjects. In particular, there were few female smokers (n = 3), therefore, the association of UA with respiratory function in female smokers could not be examined. To address this limitation, we validated the findings using 1000 replicated datasets generated by random sampling of the original dataset (Table 2). Since female smokers are at greater risk of respiratory dysfunction than male smokers [68] and we could not entirely exclude the effects of other potential confounding factors on serum UA levels, such as dietary patterns including fructose consumption and amount of daily alcohol intake, further studies in larger populations including female smokers with more detailed lifestyle information are needed to verify these findings.

Despite a number of reports on the detrimental effect of UA to the host due to its ability to be pro-oxidative under certain conditions, our data unveiled a direct beneficial connection between higher blood UA levels and maintenance of lung function in female humans and mice under aging-associated and/or COPD-associated physiopathological conditions. Because the idea of sex differences in disease susceptibility and drug responses is well-accepted, our finding of a novel concept whereby UA has a beneficial role in the lungs of postmenopausal elderly women and patients/mice suffering from aging-associated and/or COPD-associated pulmonary function decline contributes to the acceleration of sex-based medical study. 

## 5. Conclusions

Overall, our study demonstrated that blood UA directly affects lung function in female mice under pathological conditions and in human females under aging-associated physiological conditions. High plasma UA levels are mainly considered to be detrimental to the host due to the ability of UA to be pro-oxidative under certain conditions, which increases not only the risk of gout, but also many oxidative stress-related diseases. Therefore, our study provides the first unique evidence that blood UA levels directly affect lung function under certain conditions. Moreover, because the ideas on sex and life stage differences in disease susceptibility and drug responses are attracting great attention, our finding of a novel concept that UA has a beneficial role in the lungs of postmenopausal elderly women and patients/mice suffering from COPD contributes to the acceleration of sex-based medical study.

## Figures and Tables

**Table 1:** The clinical characteristics of the study subjects.

Characteristics	Males	Females	*p* Value
	(*n* = 356)	(*n* = 197)	
Age (years)	66.1 ± 9.0	66.8 ± 8.1	0.39
BMI (kg/m^2^)	23.4 ± 2.7	22.4 ± 3.2	<0.01
FEV1 / FVC (%)	75.7 ± 6.6	77.5 ± 6.2	<0.01
FEV1 (% predicted)	93.7 ± 14.0	104.1 ± 14.9	<0.01
FVC (% predicted)	105.2 ± 15.5	108.6 ± 14.7	0.01
Smoking status			
Past smoking (%)	44.4	2.5	<0.01
Current smoking (%)	15.2	0.5	
Smoking exposure (pack-years)	18.7 ± 22.8	0.3 ± 2.1	<0.01
Drinkers (%)	70.4	27.8	<0.01
Systolic BP (mmHg)	122.8 ± 15.7	120.4 ± 16.6	0.09
Diastolic BP (mmHg)	73.6 ± 11.2	70.1 ± 10.2	<0.01
LDL-C (mg/dL)	118.8 ± 26.9	124.4 ± 26.2	0.02
HDL-C (mg/dL)	63.4 ± 15.0	74.5 ± 17.8	<0.01
Triglyceride (mg/dL)	110.7 ± 63.3	95.9 ± 44.9	<0.01
Fasting plasma glucose (mg/dL)	103.7 ± 18.9	96.3 ± 14.3	<0.01
eGFR (mL/min/1.73m^2^)	69.4 ± 12.9	74.3 ± 12.7	<0.01
Uric acid (mg/dL)	5.8 ± 1.3	4.6 ± 1.0	<0.01
Hypertension (%)	46.3	36.5	0.03
Diabetes (%)	20.2	8.6	<0.01
Dyslipidemia (%)	52.8	58.4	0.21
Fatty liver (%)	24.2	14.2	<0.01
Hyperuricemia (%)	18.8	3.0	<0.01
*SLC2A9* genotype (C/C, C/T, T/T) (%)	51.1, 41.3, 7.6	48.7, 42.1, 9.1	0.73

The data are presented as the mean ± standard deviation or frequency of subjects (%). BMI: body mass index; FEV1: percent predicted forced expiratory volume in 1 second; FVC: percent of predicted forced vital capacity; BP: blood pressure; LDL-C: low-density lipoprotein cholesterol; HDL-C: high-density lipoprotein cholesterol; eGFR: estimated glomerular filtration rate; SLC2A9: solute carrier family 2 member 9.

**Table 2:** Association between serum uric acid levels and FEV1/FVC in males and females, separately.

Sex	Uric Acid	Linear Regression Analysis				Bootstrap Evaluation			
		N	B	SE	*p* value	B	SE	95% CI	*p*-Value
Males	<5 mg/dL	88	0	-	-	0	-	-	-
	5–6 mg/dL	115	1.02	0.90	0.26	1.02	0.89	−0.66, 2.82	0.26
	6–7 mg/dL	84	1.01	0.97	0.30	1.01	1.08	−1.07, 3.13	0.34
	≥7 mg/dL	69	0.28	1.07	0.79	0.28	1.07	−1.77, 2.39	0.79
Females	<4 mg/dL	58	0	-	-	0	-	-	-
	4–5 mg/dL	75	0.55	1.02	0.59	0.55	1.01	−1.47, 2.74	0.55
	5–6 mg/dL	48	0.16	1.19	0.89	0.16	1.19	−2.23, 2.63	0.89
	≥6 mg/dL	16	3.59	1.71	0.04	3.59	1.49	0.43, 6.51	0.02

B were adjusted by age, BMI, hypertension, dyslipidemia, fatty liver disease, and smoking status. B: adjusted partial regression coefficient; BMI: body mass index; FEV1: percent predicted forced expiratory volume in 1 second; FVC: percent predicted forced vital capacity; SE: standard error.

## Supplementary Materials

The following are available online at https://www.mdpi.com/2076-3921/9/5/387/s1, Figure S1: Plasma BUN and antioxidant capacity in WT and Uox-KO mice; Figure S2: The experimental scheme and plasma antioxidant capacity was higher in C57BL/6J-βENaC-Tg untreated or oxo-treated mice; Figure S3: Box plots of serum uric acid levels in males and females; Table S1: Clinical characteristics of male subjects; Table S2: Clinical characteristics of female subjects; Table S3: The association between fatty liver disease and FEV1/FVC separately in males and females.
