# Supplementary material for: Higher Blood Uric Acid in Female Humans and Mice as a Protective Factor against Pathophysiological Decline of Lung Function
Source: Antioxidants (Basel). 2020 May 6;9(5):387. doi: 10.3390/antiox9050387 (PMC7278835; doi:10.3390/antiox9050387)
Supplement: Supplementary file 1 [file antioxidants-09-00387-s001.pdf]

Supplemental Fig. S1

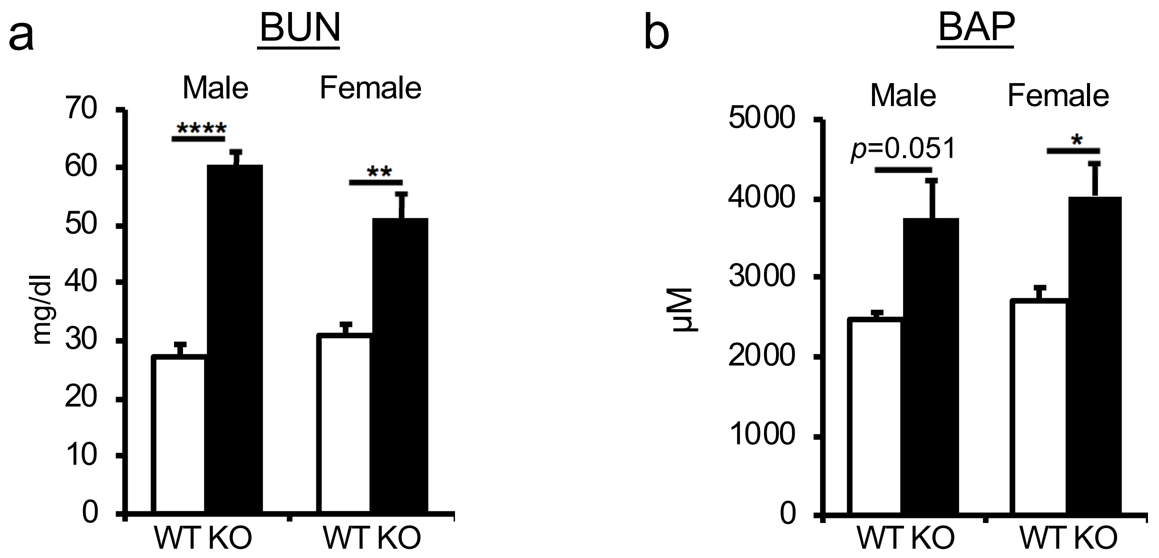

Supplementary Figure S1. Plasma BUN and antioxidant capacity in WT and Uox-KO mice. Plasma BUN level (mg/dl) (a) and antioxidant capacity (μM) (b) in male and female mice from WT and Uox-KO mice. n=3-7 mice/group. *$p<0.05$, **$p<0.001$, ****$p<0.0001$, versus WT; Student's *t*-test. Each data is expressed as mean ± SEM.

Supplemental Fig. S2

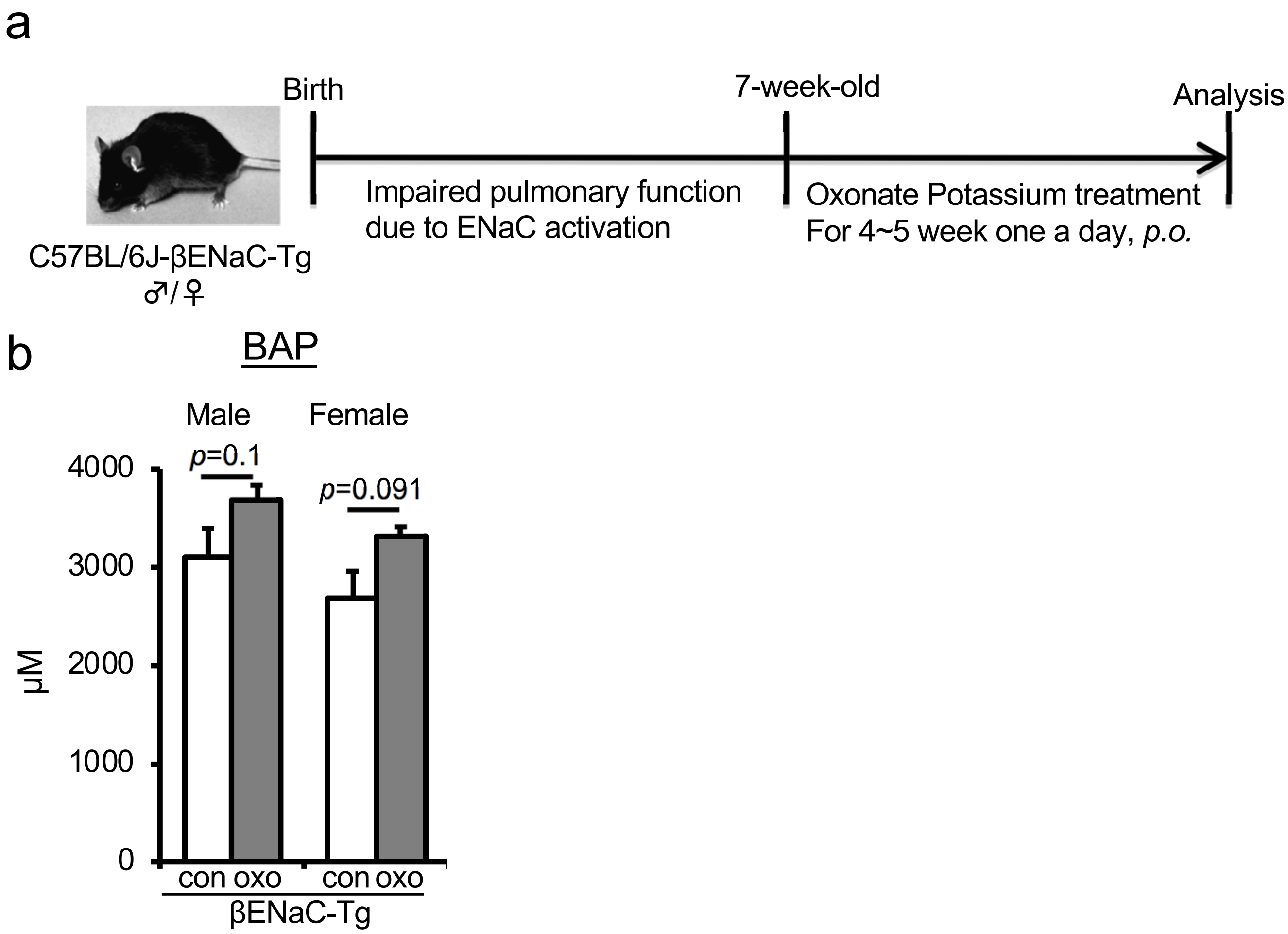

Supplementary Figure S2. The experimental scheme and plasma antioxidant capacity was higher in C57BL/6J-βENaC-Tg mice untreated or treated with oxonate. (a) The experimental scheme. (b) Plasma antioxidant capacity (μM) in male and female ENaC-Tg mice. n=4-6 mice/group. *$p<0.05$, **$p<0.001$, ****$p<0.0001$, versus WT; Student's *t*-test. The data is expressed as mean ± SEM.

Supplemental Fig. S3

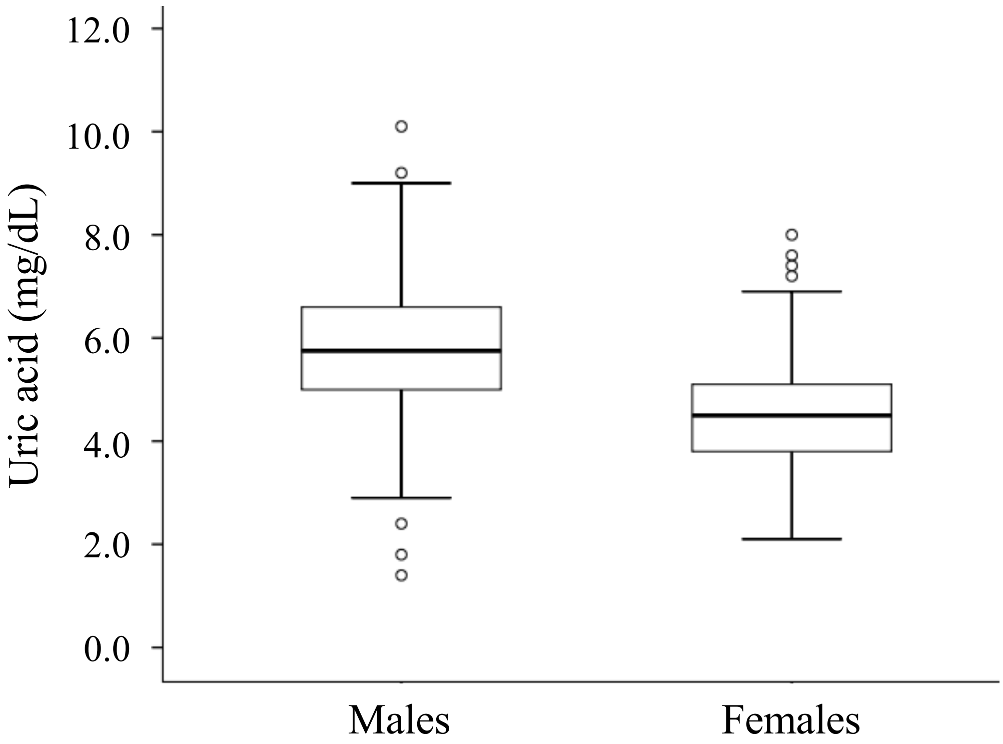

Supplementary Figure S3. The box plots of serum uric acid levels in males and females.

Supplementary Table S1. The clinical characteristics of the male subjects.

Characteristics	Uric acid				*P* value
	**<5 mg/dL (n = 88)**	**5-6 mg/dL (n = 115)**	**6-7 mg/dL (n = 84)**	**≥7 mg/dL (n = 69)**	
Age (years)	67.9 ± 8.6	66.6 ± 9.3	66.8 ± 8.6	62.2 ± 8.3	< 0.01
BMI (kg/m^2)	22.7 ± 3.0	23.1 ± 2.5	23.7 ± 2.6	24.4 ± 2.4	< 0.01
FEV1 / FVC (%)	74.6 ± 6.5	76.0 ± 6.3	75.9 ± 7.3	76.3 ± 6.5	0.34
FEV1 (% predicted)	90.4 ± 14.5	96.3 ± 14.1	95.4 ± 13.0	91.4 ± 13.4	< 0.01
FVC (% predicted)	101.5 ± 14.7	107.2 ± 14.7	106.9 ± 17.1	104.8 ± 15.0	0.04
Smoking status					
Past smoking (%)	39.8	48.7	42.9	44.9	0.73
Current smoking (%)	15.9	11.3	16.7	18.8	
Smoking exposure (pack-years)	17.8 ± 22.6	18.0 ± 20.8	18.4 ± 24.5	21.6 ± 24.4	0.70
Drinkers (%)	52.9	74.8	73.8	81.2	< 0.01
Systolic BP (mmHg)	121.3 ± 13.5	122.8 ± 16.3	122.6 ± 15.4	124.7 ± 17.4	0.60
Diastolic BP (mmHg)	71.4 ± 10.1	73.1 ± 11.2	73.2 ± 11.5	77.4 ± 11.5	< 0.01
LDL-C (mg/dL)	114.2 ± 26.3	120.6 ± 27.8	115.2 ± 23.8	126.1 ± 28.5	0.02
HDL-C (mg/dL)	66.4 ± 14.8	64.8 ± 16.1	61.5 ± 13.0	59.4 ± 15.0	0.01
Triglyceride (mg/dL)	99.6 ± 58.8	98.8 ± 52.6	112.6 ± 61.0	142.2 ± 76.8	< 0.01

Fasting plasma glucose (mg/dL)	104.9 ± 24.4	103.8 ± 20.0	102.9 ± 12.7	103.0 ± 15.6	0.90
eGFR (mL/min/1.73m^2)	75.0 ± 13.6	71.3 ± 11.4	66.6 ± 10.6	62.4 ± 12.9	< 0.01
Hypertension (%)	40.9	44.3	48.8	53.6	0.41
Diabetes (%)	22.7	15.7	25.0	18.8	0.37
Dyslipidemia (%)	46.6	48.7	54.8	65.2	0.09
Fatty liver (%)	15.9	21.7	25.0	37.7	0.02
SLC2A9 genotype (C/C, C/T, T/T) (%)	55.7, 38.6, 5.7	48.7, 41.7, 9.6	51.2, 44.0, 4.8	49.3, 40.6, 10.1	0.78

The data are presented as the mean ± standard deviation or frequency of subjects (%)

BMI, body mass index; FEV1: percent predicted forced expiratory volume in 1 second; FVC, percent predicted forced vital capacity; BP, blood pressure; LDL-C, low-density lipoprotein cholesterol; HDL-C, high-density lipoprotein cholesterol; eGFR, estimated glomerular filtration rate; SLC2A9, solute carrier family 2 member 9.

Supplementary Table S2. The clinical characteristics of the female subjects.

Characteristics	Uric acid				*P* value
	**<4 mg/dL (n = 58)**	**4-5 mg/dL (n = 75)**	**5-6 mg/dL (n = 48)**	**≥6 mg/dL (n = 16)**	
Age (years)	66.0 ± 7.9	66.8 ± 7.9	67.5 ± 8.0	67.3 ± 10.6	0.81
BMI (kg/m²)	21.3 ± 2.5	21.9 ± 2.7	23.8 ± 3.6	24.6 ± 3.7	< 0.01
FEV1 / FVC (%)	77.5 ± 6.4	77.6 ± 6.2	76.7 ± 6.4	79.8 ± 5.4	0.38
FEV1 (% predicted)	105.8 ± 13.0	103.8 ± 15.5	102.5 ± 17.3	104.3 ± 10.5	0.73
FVC (% predicted)	111.2 ± 14.6	108.3 ± 14.6	107.2 ± 16.7	105.4 ± 8.1	0.40
Smoking status					
Past smoking (%)	1.7	2.7	2.1	6.3	0.67
Current smoking (%)	1.7	0.0	0.0	0.0	
Smoking exposure (pack-years)	0.3 ± 2.6	0.4 ± 2.4	0.0 ± 0.0	0.4 ± 1.5	0.76
Drinkers (%)	24.1	28.0	33.3	25.0	0.76
Systolic BP (mmHg)	119.7 ± 17.6	121.9 ± 15.4	117.5 ± 17.7	123.9 ± 15.4	0.41
Diastolic BP (mmHg)	69.0 ± 9.8	72.0 ± 9.7	68.4 ± 10.0	70.1 ± 13.3	0.21
LDL-C (mg/dL)	121.0 ± 26.5	124.8 ± 27.1	125.3 ± 25.1	132.6 ± 23.9	0.46
HDL-C (mg/dL)	77.7 ± 17.1	75.5 ± 18.6	69.7 ± 17.1	72.9 ± 17.1	0.13
Triglyceride (mg/dL)	84.4 ± 45.4	96.4 ± 44.1	106.6 ± 46.6	103.1 ± 34.3	0.07

Fasting plasma glucose (mg/dL)	95.5 ± 14.4	96.8 ± 16.7	97.4 ± 11.3	93.5 ± 9.7	0.77
eGFR (mL/min/1.73m^2)	80.6 ± 10.9	72.9 ± 11.2	72.1 ± 12.1	63.9 ± 16.9	< 0.01
Hypertension (%)	25.9	36.0	43.8	53.8	0.08
Diabetes (%)	6.9	13.3	6.3	0.0	0.33
Dyslipidemia (%)	44.8	64.0	64.6	62.5	0.10
Fatty liver (%)	5.2	12.0	25.0	25.0	0.01
SLC2A9 genotype (C/C, C/T, T/T) (%)	51.7, 43.1, 5.2	52.0, 40.0, 8.0	45.8, 43.8, 10.4	31.3, 43.8, 25.0	0.37

The data are presented as the mean ± standard deviation or frequency of subjects (%)
BMI, body mass index; FEV1: percent predicted forced expiratory volume in 1 second; FVC, percent predicted forced vital capacity; BP, blood pressure; LDL-C, low-density lipoprotein cholesterol; HDL-C, high-density lipoprotein cholesterol; eGFR, estimated glomerular filtration rate; SLC2A9, solute carrier family 2 member 9.

Supplementary Table S3. The association between fatty liver disease and FEV1/FVC separately in males and females.

Sex	Model	Covariate	Linear regression analysis		
			**B**	**SE**	***P* value**
Males	1	Presence of fatty liver disease	1.97	0.82	0.02
	2	Presence of fatty liver disease	1.05	0.80	0.19
		Per 1-year increase in age	-0.22	0.04	< 0.01
Females	1	Presence of fatty liver disease	1.26	1.27	0.33
	2	Presence of fatty liver disease	0.51	1.18	0.67
		Per 1-year increase in age	-0.30	0.05	< 0.01

B, adjusted partial regression coefficient; FEV1, percent predicted forced expiratory volume in 1 second; FVC, percent predicted forced vital capacity; SE, standard error.
